# High Biologically Effective Dose Radiotherapy for Brain Metastases May Improve Survival and Decrease Risk for Local Relapse Among Patients With Small-Cell Lung Cancer: A Propensity-Matching Analysis

**DOI:** 10.1177/1073274820936287

**Published:** 2020-07-02

**Authors:** Qing-yang Zhuang, Jin-luan Li, Fei-fei Lin, Xi-jin Lin, Huaqin -lin, Youjia -Wang, Yaobin -Lin, Yun-xia Huang, Xue-qing Zhang, Li-rui Tang, Jun-xin Wu

**Affiliations:** 1 Department of Radiation Oncology, Fujian Cancer Hospital, Fujian Medical University Cancer Hospital, Fuzhou, China.; 2 Department of Radiation Oncology, Xiamen Cancer Center, The First Affiliated Hospital, School of Medicine, Xiamen University, Teaching Hospital of Fujian Medical University, Xiamen, People’s Republic of China; 3 Department of Renal Cancer and Melanoma, The Key Laboratory of Carcinogenesis and Translational Research (Ministry of Education), Peking University Cancer Hospital and Institute, Beijing, People’s Republic of China

**Keywords:** biologically effective dose, radiotherapy, propensity score, small-cell lung cancer, brain metastasis, overall survival, progression-free survival

## Abstract

To evaluate whether high biologically effective dose (BED) radiotherapy improves local control and survival outcomes for patients with brain metastases (BMs) from small-cell lung cancer (SCLC) and to determine possible prognostic factors. From January 1998 to June 2018, 250 patients with BM from SCLC were retrospectively analyzed. The Cutoff Finder program was used to classify patients by BED. Overall survival (OS) and BM progression-free survival (BM-PFS) were analyzed using the Kaplan-Meier method and log-rank test. A Cox regression model was used to calculate the hazard ratio and 95% CI for prognostic factors for OS among the study population and propensity score (PS)–matched patients. A BED of 47.4 was taken as the optimal cutoff value. Both OS and BM-PFS were significantly improved in the high-BED (>47.4 Gy) than in the low-BED (≤47.4 Gy) group (median OS: 17.5 months vs 9.5 months, *P* < .001, median BM-PFS: 14.4 months vs 8.3 months, *P* < .001). Biologically effective dose (*P* < .001), Eastern Cooperative Oncology Group performance status (*P* = .047), smoking (*P* = .005), and pleural effusion (*P* = .004) were independent prognostic factors for OS. Propensity score matching with a ratio of 1:2 resulted in 57 patients in the high-BED group and 106 patients in the low-BED group. In the PS-matched cohort, OS and BM-PFS were significantly prolonged in the high-BED group compared with the low-BED group (*P* < .001). Biologically effective dose >47.4 Gy improves survival among patients with BM from SCLC. Eastern Cooperative Oncology Group score, smoking, and pleural effusion independently affect OS of SCLC patients with BM.

## Background

Small-cell lung cancer (SCLC) is decreasing in incidence in developed countries, likely reflecting the declining popularity of cigarette smoking, particularly in men.^1^ Small-cell lung cancer is very aggressive, characterized by early dissemination and eventual metastasis, with an overall 5-year relative survival rate of approximately 19%.^2^ Brain metastasis (BM) is the most common and devastating complication of SCLC.^3^ Approximately 50% to 80% of patients will develop BM within the first 2 years after the initial diagnosis of SCLC.^4^ Small-cell lung cancer with active BM has a dismal prognosis with high morbidity and mortality. Effective therapeutic approaches to benefit survival and quality of life with BM are, therefore, a clinical priority.

For patients with BM, the standard treatment recommended by the National Comprehensive Cancer Network guidelines is whole-brain radiotherapy (WBRT).^5^ However, the persistence or progression of BM treated with prophylactic cranial irradiation or WBRT is frequently observed. The administration of a local radiation boost such as stereotactic radiosurgery (SRS) is therefore suggested. This approach has been shown to be safe and effective for local tumor control.^6^ The standard dose of WBRT alone is 30 Gy in 10 fractions,^7^ but the optimal radiation dose of WBRT when used in combination with a radiation boost for BM from SCLC has not yet been standardized. A recent study of 82 patients with BM from SCLC suggested that the addition of a radiation boost for patients who received WBRT improves overall survival (OS), but the study did not specifically address the dose–response relationship.^8^ Administering a peripheral biologically effective dose (BED) of 80 Gy during SRS resulted in local control of 94.5% at 1 year for BM from non-small cell lung cancer (NSCLC), suggesting the potential therapeutic use of BED-based radiotherapy for BM control.^9^ A recent systematic review reported that increases in BED were correlated with improved OS and decreased risk of local relapse in patients with limited-stage SCLC, indicating a dose–response benefit of BED and supporting the use of a radiation dose-escalation strategy in SCLC treatment.^10^ To date, no standardized strategy for escalation of the BED during brain radiotherapy has been established for patients with SCLC.^11^

Very few studies have been launched so far to elucidate and decipher BED with meaningful clinical benefit for the metastatic brain tumors. Although several studies have been performed to elucidate BED in predicting the outcomes of NSCLC after radiotherapy, the prognostic value of BED to the brain for patients with SCLC has not been well addressed. No consensus has been reached with regard to histological type-specific prognostic factors after radiotherapy to the brain. Therefore, the possible prognostic importance of BED in brain radiotherapy was retrospectively evaluated in a cohort of patients with SCLC and BM.

## Materials and Methods

### Patient Selection and Data Extraction

A total of 250 consecutive patients with SCLC and BMs treated at our institution between June 1998 and June 2018 were retrospectively studied. The study was approved by the ethics committee of our hospital (no KT2018-004-01), and informed consent was waived due to the retrospective nature of the study. Eligibility required pathological or cytological confirmation of a primary SCLC, the presence of BM on magnetic resonance imaging (MRI) and/or computed tomography (CT), at least 1 radiotherapy regimen for BM, no previous or concurrent diagnosis of other malignancies, and at least 1 follow-up visit after radiotherapy for BM.

The clinical records of eligible patients were reviewed and the following baseline variables were retrieved: age, gender, smoking status, Eastern Cooperative Oncology Group (ECOG) performance status, clinical stage (American Joint Committee on Cancer eighth edition), extent of disease, pleural effusion, external metastasis and the number of involved organs, time interval from the diagnosis of primary SCLC to BM, surgery on the primary tumor, chemotherapy and radiotherapy regimens, and BED to the brain. Additional variables relevant to BM included the number of brain tumors and the maximum diameter of the largest tumor, bilateral BM, subfalcine herniation, severe symptoms, and diagnosis-specific graded prognostic assessment (GPA) score. The diagnosis of BM was performed by 2 independent radiologists using MRI/CT images of the brain and clinical manifestations.

### Treatment and BED

Among 250 eligible patients, 225 received concurrent or sequential chemotherapy, and 132 received radiotherapy for the primary disease after receiving a diagnosis of SCLC. All 250 patients received 6-MV photon beams for BM: 208 patients received WBRT alone; 42 patients met the following criteria were considered for a boost: (1) ECOG Performance Status score ≤2, (2) well-controlled primary disease, and (3) number of BMs lesions ≤5. Of the 208 patients receiving WBRT only, 22 did not complete the course of treatment due to personal choice. In the WBRT-alone group, 203 patients were treated with conventional 2-dimensional radiotherapy (2D-RT); the other 5 patients were treated with intensity-modulated radiation therapy (IMRT). The median dose was 30 Gy (range 3-50 Gy). The median cumulative BED was 39 Gy (range 3.9-67.2 Gy). Among them, 33 patients received BED more than 47.4 Gy. For these 208 patients, individually shaped shielding blocks were fabricated when necessary. Radiotherapy was performed using a minimum source-to-skin distance as dictated by tumor size and location. For IMRT, gross tumor volume (GTV) encompassing contrast-enhancing tumors was calculated by radiation oncologists and neurosurgeons based on tumor volume and location as measured in MRI and relevant neurological symptoms. The margin from GTV to planning target volume was 1 to 2 mm. In the WBRT plus boost group, 15 patients received 2D-RT, 25 patients received IMRT, and WBRT plus SRT (stereotactic radiotherapy) was administered in 1 patient. The median dose was 45 Gy (range 35-70 Gy). The median cumulative BED of the grossly metastatic tumor was 58.5 Gy (range 41.1-98.2 Gy). Among them, 27 patients received BED more than 47.4 Gy.

Given that various dose-fractionation regimens were applied, we used the concept of BED to facilitate comparisons of these radiotherapy regimens. With the consideration of treatment time and redistribution of cell cycles, the BED was calculated with the linear quadratic approach $\left(\text{BED}=n\times d\left(1\;+\frac{\text{d}}{\frac{\text{α}}{\text{β}}}-\frac{\text{α}}{\text{γ}}\times (T-Tk\right)\frac{\text{α}}{\text{γ}},\frac{\text{α}}{\text{γ}}=\frac{0.6Gy}{d}\right)$, *Tk* = 7 days, *T* = total number of treatment days elapsed) and assuming the αβ ratio to be 10 Gy for the brain (BED_10_).^12,13^ The total BED ranged from 3.9 to 98.2 Gy (median 39.0 Gy).

### Study End Points

The primary end points were OS and BM progression-free survival (BM-PFS). Overall survival was defined as the interval from initial BM diagnosis to death due to any cause. The BM-PFS was the interval between the diagnosis of BM and the earlier of BM progression or death due to any cause.^14^ Brain metastasis progression was confirmed by MRI and/or CT. Patients alive at the last follow-up were censored. There was no loss to follow-up.

### Statistical Analysis

All statistical analyses were carried out in SPSS version 23.0 software (IBM Corp). The optimal cutoff value for BED was ascertained using the Cutoff Finder online tool (molpath.charite.de/cutoff).^15^ The Kaplan-Meier method and log-rank test were used for survival analysis comparing patients with high and low BEDs. The χ^2^ test was used to analyze differences in categorical variables. The Cox proportional hazards regression model was used to identify prognostic factors for OS in univariate and multivariate analyses. The resulting data were reported as hazard ratio (HR) with 95% CI. Propensity score (PS) matching was implemented using SPSS software with the PS Matching 3.0.4 plugin. To control for selection bias, PS matching with caliper width standard deviation of 0.2 was conducted at a 1:2 ratio based on the following covariates: number of BMs, laterality of BM, symptomatic BM, and interval between the diagnosis of SCLC and BM. Two-sided *P* values of less than .05 were considered to be statistically significant.

## Results

### Patient Characteristics

Two hundred fifty patients meeting the inclusion criteria were included in this study, comprising of 234 (93.6%) males and 16 (6.4%) females. Table 1 presents the characteristics of these patients at baseline. One hundred sixty-six (66.4%) patients reported a history of smoking; 84 (33.6%) were nonsmokers. Median age at diagnosis of BM was 59 (range 33-79) years; 227 (90.8%) patients had an ECOG performance status score of 0; 154 (61.6%) patients had a GPA score >1.5. At the time of the initial SCLC diagnosis, 16 (6.4%) patients were at stage II; 234 (93.6%) were at stage III or IV. Sixty-four (25.6%) patients had a pleural effusion based on the results of chest imaging. Most (83.2%) patients developed metastases to external sites other than the brain, including the liver, kidney, bone, and adrenal gland; 37 (14.8%) had metastases in more than 2 organs. Most patients (225, 90%) received chemotherapy for the primary disease after receiving an initial diagnosis of SCLC; 24 (9.6%) received ≥10 cycles. One hundred thirty-two (47.2%) patients were treated with chest radiotherapy to the primary site.

**Table 1.** Baseline Characteristics of 250 Patients With Small-Cell Lung Cancer and Brain Metastasis.

Variables	N	%
Age (years)		
<60	125	50
≥60	125	50
ECOG score		
0	227	90.8
1	19	7.6
2	2	0.8
3	2	0.8
Gender		
Male	234	93.6
Female	16	6.4
Smoking		
Yes	166	66.4
No	84	33.6
Clinical stage		
II	16	6.4
III + IV	234	93.6
Surgery in primary lesion		
Yes	17	6.8
No	233	93.2
Chemotherapy before BM		
Yes	225	90
No	25	10
Chemo cycles		
<10	226	90.4
≥10	24	9.6
Radiotherapy in primary lesion		
Yes	132	47.2
No	118	52.8
BED in brain		
≤47.4 Gy	190	76
>47.4 Gy	60	24
RFI (months)		
≤20	232	92.8
>20	18	7.2
External metastasis		
Yes	208	83.2
No	42	16.8
Number of external metastasis		
≤2	213	85.2
>2	37	14.8
Maximum diameter of the largest tumor (cm)		
≤2	112	44.8
2-4	100	40
4-6	25	10
≥6	13	5.2
Bilateral BM		
Yes	134	53.6
No	116	46.4
Subfalcine herniation		
Yes	17	6.8
No	233	93.2
Number of BMs		
≤3	105	42
>3	145	58
Severe neurological symptom		
Yes	53	78.8
No	197	21.2
Pleural effusion		
Yes	64	25.6
No	186	74.4
GPA		
≤1.5	96	38.4
>1.5	154	61.6

Abbreviations: BED, biologically effective dose; BM, brain metastasis; ECOG, Eastern Cooperative Oncology Group; GPA, graded prognostic assessment; RFI, recurrence-free interval.

At the time of BM diagnosis, 105 (42%) patients had 3 or fewer BMs; 145 (58%) had >3 BMs. In terms of the maximum diameter of the largest BM, 122 (44.8%) patients had maximum tumor size <2 cm; 100 (40%) had maximum tumor size of 2 to 4 cm; 25 (10%) had maximum tumor size of 4 to 6 cm; 13 (5.2%) had maximum tumor size >6 cm. Bilateral and unilateral BMs were observed in 134 (53.6%) and 116 (46.4%) patients, respectively. Seventeen (6.8%) patients were diagnosed with subfalcine herniation, and 53 (6.0%) patients experienced severe symptoms including memory loss, walking/gait disorder, and blurry vision.

### Biologically Effective Dose Cutoff Optimization

**Figure 1.**
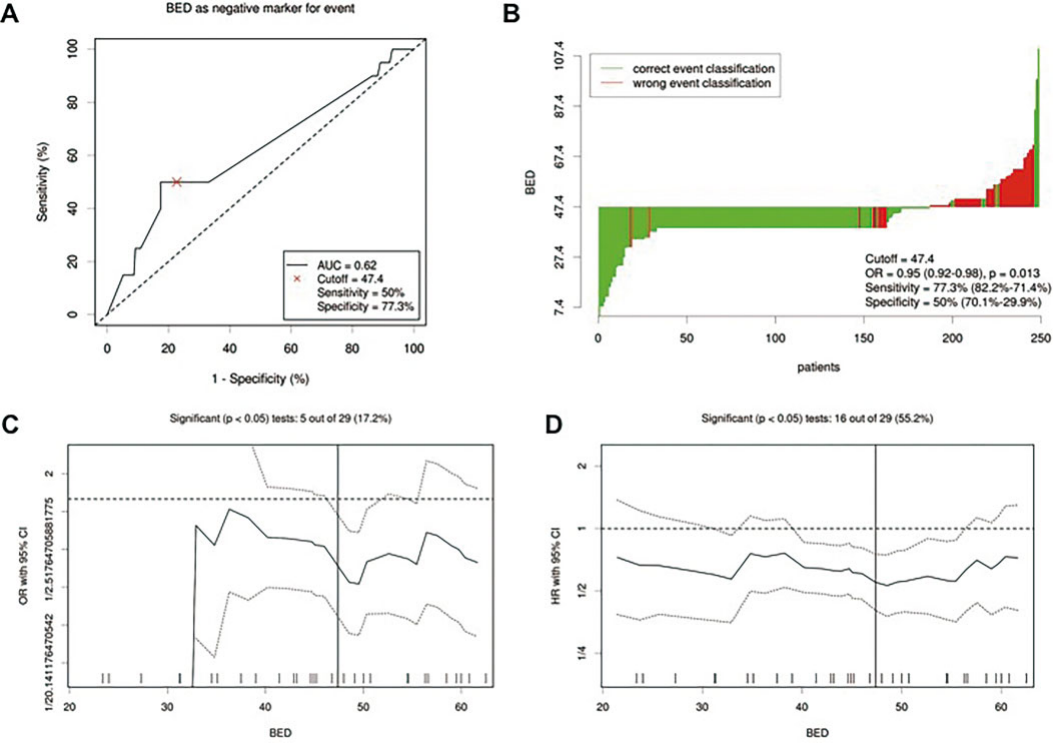
Distribution-based cutoff optimization of biologically effective dose (BED) in patients with brain metastasis (BM) from small-cell lung cancer (SCLC). A, Receiver operating characteristic curve for BED. Biologically effective dose value was used as a negative marker for the event of death. A BED score of 47.4 was chosen as the cutoff point representing the optimal balance between sensitivity (50%) and specificity (77.3%). Area under the curve = 0.62. B, Waterfall plot of BED. Biologically effective dose values were stratified with the optimal threshold obtained from (A). C, Plot of the odds ratio with 95% CI for each BED threshold. D, Plot of the hazard ratio with 95% CI for each BED threshold. Vertical lines indicate the data distribution correlated most strongly with overall survival. Dashed lines indicate the 95% CIs. The distribution of BED values among the 250 patients included in this study is shown as a rug plot at the bottom of the figure.

With use of the Cutoff Finder algorithm, 47.4 Gy (such as 40 Gy with 2 Gy per fraction for whole-brain radiation) was taken as the optimal cutoff value for BED in all patients before PS matching (Figure 1). Of 250 patients, 190 (76%) patients had BED ≤ 47.4 Gy (low-BED group) and 60 (24%) patients had BED > 47.4 Gy (high-BED group).

### Propensity Score Matching

Propensity score matching was performed with a ratio of 1:2 and covariate adjustment to minimize clinical differences between patients who received brain radiotherapy at low- and high-BED levels.^16^ Propensity score matching yielded 57 and 106 patients in the high- and low-BED group, respectively. Table 2 presents the demographic and clinical differences between patients with different BED levels before and after PS matching, with no significant difference observed between PS-matched groups (*P*s > .1).

**Table 2.** Characteristics of Patients With Small-Cell Lung Cancer and Brain Metastasis Stratified by BED Before and After Propensity Score Matching.

Variables	Before matching			After matching		
	BED ≤ 47.4 Gy (n)	BED > 47.4 Gy (n)	*P*	BED ≤ 47.4 Gy (n)	BED > 47.4 Gy (n)	*P*
Age (years)			.882			.870
<60/≥60	94/96	31/29		56/50	29/28	
ECOG score			.723			.531
0/1/2/3	172/14/2/2	55/5/0/0		93/9/2/2	52/5/0/0	
Gender			.545			1.000
Male/female	179/11	55/5		98/8	53/4	
Smoking			.638			.796
Yes/no	128/62	38/22		36/70	20/37	
Clinical stage			.545			.518
II/III-IV	11/179	5/55		6/100	5/52	
Surgery in primary lesion			.565			1.000
Yes/no	12/178	5/55		8/98	4/53	
Chemotherapy before BMs			.215			.263
Yes/no	168/22	57/3		94/12	54/3	
Chemo cycles						
<10/≥10	171/19	55/5	.806	95/11	52/5	1.000
Radiotherapy in primary lesion			.138			.254
Yes/no	95/95	37/23		55/51	35/22	
RFI (months)			**.046**			**.518**
≤20/>20	180/10	52/8		100/6	52/5	
External metastasis			.299			.739
Yes/no	92/98	24/36		45/61	22/35	
Number of external metastasis			.058			.299
≤2/>2	157/33	56/4		92/14	53/4	
Maximum diameter of the largest tumor (cm)			.576			.858
<2/2-4/4-6/>6	82/80/17/10	29/20/8/3		45/43/11/7	28/20/6/3	
Bilateral brain metastasis			**.001**			**.818**
Yes/no	113/77	21/39		41/65	21/36	
Subfalcine herniation			.565			.518
Yes/no	12/178	5/55		6/100	5/52	
Number of BMs			**.001**			**1.000**
≤3/>3	68/122	37/23		64/42	34/23	
Severe neurological symptoms			**.030**			**.572**
Yes/no	34/156	19/41		25/81	16/41	
Pleural effusion			.612			1.000
Yes/no	47/143	17/43		28/78	15/42	
GPA			.763			.608
≤1.5/>1.5	72/118	24/36		36/70	22/35	

Abbreviations: BED, biologically effective dose; BM, brain metastasis; ECOG, Eastern Cooperative Oncology Group; GPA, graded prognostic assessment; RFI, recurrence-free interval.

The bold values indicate P-value is less than 0.05.

### Overall Survival and BM-PFS

**Figure 2.**
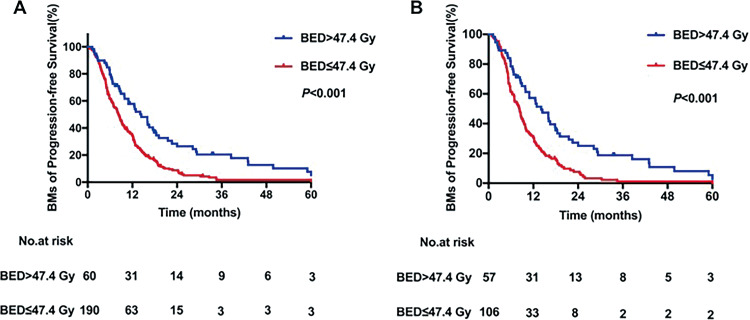
Kaplan-Meier curves for overall survival stratified by biologically effective dose (A) for peer review before and (B) after propensity score matching.

The median duration of follow-up was 18.9 (range, 0.5-207.3) months. Overall, death occurred in 180 (94.7%) patients in the low-BED group and 50 (83.3%) patients in the high-BED group during the follow-up; all deaths were cancer-related. In the high-BED group, the median OS was 17.5 months, with 1-, 3-, and 5-year OS rates of 70.88%, 22.58%, and 5.02%, respectively. In the low-BED group, the median OS was 9.5 months, with 1-, 3-, and 5-year OS rates of 41.72%, 2.79%, and 1.34%, respectively. The difference in OS between high- and low-BED groups was significant (*P* < .001, Figure 2A). The results were similar after PS matching, showing significantly worse OS in the low-BED group than the high-BED group (*P* < .001, Figure 2B).

**Figure 3.**
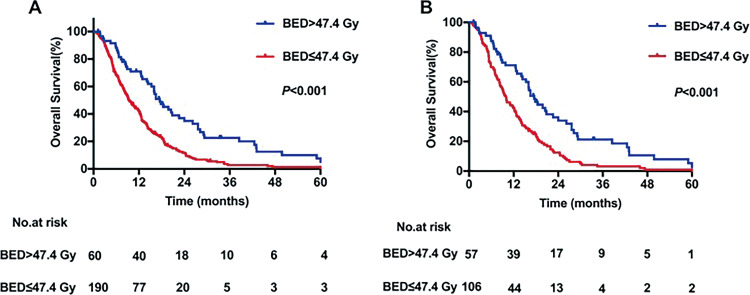
Kaplan-Meier curves for brain metastasis progression-free survival ( stratified by biologically effective dose (A) before and (B) after propensity score matching.

Median BM-PFS among the entire cohort was 8.6 (range, 0.3-207.3) months. The BM-PFS was significantly increased in patients who were treated with high-BED radiotherapy to the brain as compared with patients who were treated with low-BED radiotherapy to the brain (*P* < .001, Figure 3A). The 1-, 3-, and 5-year BM-PFS rates in the high-BED group were 57.84%, 20.35%, and 5.09%, respectively, while in the low-BED group, the corresponding BM-PFS rates were 34.19%, 1.71%, and 1.71%, respectively. Similar results were obtained in PS-matched patients, with 1-, 3-, and 5-year BM-PFS rates of 57.31%, 18.8%, and 5.38%, respectively, in the high-BED group and 30.89%, 1.09%, and 1.09%, respectively, in the low-BED group. The difference between groups was significant for each time point (*P* < .001, Figure 3B).

### Univariate and Multivariate Cox Regression Analysis for OS

Table 3 presents the results of univariate and multivariate analyses of factors affecting OS in all patients before PS matching. In multivariate analysis, higher ECOG score was correlated with worse OS (*P* = .042), while age ≥60 years (HR = 0.671, 95% CI: 0.511-0.882, *P* < .004), nonsmoker status (HR = 0.739, 95% CI: 0.559-0.976, *P* = .033), and BED >47.4 Gy (HR = 0.383, 95% CI: 0.261-0.564, *P* < .001) were independent prognostic factors associated with improved OS. External metastasis and number of BMs were significantly associated with OS in univariate analysis, but the significance disappeared in multivariate analysis.

**Table 3.** Cox Regression Analysis of 250 Patients With Small-Cell Lung Cancer and Brain Metastasis.

Variable	n	Univariate			Multivariate		
		HR	95% CI	*P*	HR	95% CI	*P*
Age (years)							
<60/≥60	125/125	0.763	0.558-0.990	.042	0.671	0.511-0.882	**.004**
ECOG score							
0	227			.021			**.042**
1	19	1.304	0.814-2.090	.270	1.415	0.849-2.358	.183
2	2	4.860	1.184-19.955	.028	4.006	0.941-17.053	.060
3	2	4.426	1.078-18.173	.039	3.941	0.934-16.637	.062
Gender							
Male/female	234/16	1.557	0.934-2.597	.089			
Smoking							
Yes/no	166/84	0.719	0.548-0.943	.017	0.739	0.559-0.976	**.033**
Clinical stage							
II/III-IV	16/234	1.607	0.931-2.773	.089			
Surgery in primary lesion							
Yes/no	17/233	0.707	0.423-1.181	.185			
Chemotherapy before BM							
Yes/no	225/25	1.138	0.750-1.728	.542			
Chemo cycles							
<10/≥10	226/24	0.995	0.628-1.576	.983			
Radiotherapy in primary lesion							
Yes/no	132/118	0.790	0.608-1.027	.078			
BED in brain							
≤47.4 Gy/ >47.4 Gy	190/60	0.478	0.346-0.660	<.001	0.383	0.261-0.564	**<.001**
RFI (months)							
≤20/>20	18/232	1.161	0.872-1.547	.307			
External metastasis							
Yes/no	208/42	1.301	1.004-1.686	.047			
Number of external metastasis							
≤2/>2	213/37	1.209	0.846-1.726	.298			
Maximum diameter of the largest tumor (cm)							
<2	112			.164			
2-4	100	1.181	0.890-1.566	.249			
4-6	25	0.674	0.411-1.103	.116			
>6	13	1.012	0.566-1.811	.968			
Bilateral BM							
Yes/no	134/116	1.081	0.833-1.403	.557			
Subfalcine herniation							
Yes/no	17/233	1.039	0.633-1.707	.879			
Number of BMs							
≤3/>3	105/145	1.322	1.015-1.723	.039			
Severe neurological symptom							
Yes/no	53/197	0.902	0.657-1.237	.522			
Pleural effusion							
Yes/no	64/186	1.192	0.877-1.620	.262	1.336	0.958-1.862	.088
GPA							
≤1.5/>1.5	96/154	0.915	0.702-1.192	.511			

Abbreviations: BED, biologically effective dose; BM, brain metastasis; ECOG, Eastern Cooperative Oncology Group; GPA, graded prognostic assessment; HR, hazard ratio; RFI, recurrence-free interval; SCLC, small-cell lung cancer.

The bold values indicate P-value is less than 0.05.

**Figure 4.**
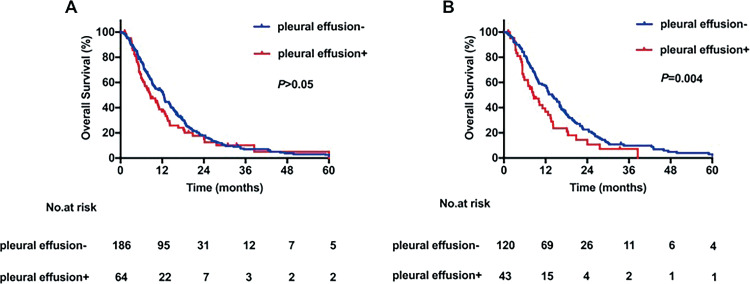
Kaplan-Meier curves for overall survival stratified by the presence of pleural effusion (A) for peer review before and (B) after propensity score matching.

Table 4 summarizes the results of univariate and multivariate survival analyses of OS in PS-matched patients. Similar to the results obtained from the entire study group, high ECOG score and smoking history were independent prognostic factors of worse OS in patients with SCLC and BM. A BED > 47.4 Gy remained a prognostic factor strongly and independently associated with better OS (HR = 0.419, 95% CI: 0.284-0.618, *P* < .001). Notably, in PS-matched patients, pleural effusion was found to be independently associated with OS (HR = 1.838, 95% CI: 1.217-2.777, *P* = .004). Analysis of group differences with the log-rank test revealed better OS in patients without pleural effusion compared with those with pleural effusion (1-year OS: 55.7% vs 36.8%) after PS matching; analysis performed before PS matching revealed no significant difference (Figure 4).

**Table 4.** Cox Regression Analysis of 163 Propensity Matched Patients With Small-Cell Lung Cancer and Brain Metastasis.

Variable	n	Univariate			Multivariate		
		HR	95% CI	*P*	HR	95% CI	*P*
Age (years)							
<60/≥60	85/78	0.750	0.544-1.034	.079	0.736	0.519-1.043	.085
ECOG score							
0	145			.009			**.047**
1	14	1.316	0.757-2.289	.330	1.542	0.862-2.759	.145
2	2	6.021	1.433-25.297	.014	3.726	0.822-16.887	.088
3	2	5.708	1.358-23.993	.017	4.257	0.963-18.823	.056
Gender							
Male/female	151/12	1.402	0.773-2.542	.266			
Smoking							
Yes/no	56/107	0.607	0.433-0.850	.004	0.601	0.422-0.855	**.005**
Clinical stage							
II/III-IV	11/152	1.767	0.893-3.495	.102			
Surgery in primary lesion							
Yes/no	12/151	0.583	0.312-1.088	.090			
Chemotherapy before BMs							
Yes/no	148/15	1.165	0.679-1.998	.579			
Chemo cycles							
<10/≥10	147/16	1.105	0.647-1.888	.715			
Radiotherapy in primary lesion							
Yes/no	90/73	0.800	0.577-1.110	.182			
BED in brain							
≤47.4 Gy/>47.4 Gy	106/57	0.501	0.353-0.711	<.001	0.419	0.284-0.618	**<.001**
RFI (months)							
≤20/>20	152/11	0.923	0.485-1.757	.808			
External metastasis							
Yes/no	67/96	1.196	0.865-1.654	.279			
Number of external metastasis							
≤2/>2	96/67	1.132	0.689-1.862	.624			
Maximum diameter of the largest tumor (cm)							
<2	73			.470			
2-4	63	1.202	0.845-1.710	.306			
4-6	17	0.803	0.456-1.414	.447			
≥6	10	1.242	0.634-2.433	.527			
Bilateral brain metastasis							
Yes/no	62/101	0.850	0.611-1.182	.333			
Subfalcine herniation							
Yes/no	11/152	0.881	0.474-1.637	.688			
Number of BMs							
≤3/>3	98/65	1.096	0.787-1.526	.588			
Severe neurological symptom							
Yes/no	41/122	0.987	0.684-1.423	.943			
Pleural effusion							
Yes/no	43/120	1.524	1.044-2.225	.029	1.838	1.217-2.777	**.004**
GPA							
≤1.5/>1.5	105/58	0.937	0.672-1.306	.702			

Abbreviations: BED, biologically effective dose; BM, brain metastasis; ECOG, Eastern Cooperative Oncology Group; GPA, graded prognostic assessment; HR, hazard ratio; RFI, recurrence-free interval; SCLC, small-cell lung cancer.

The bold values indicate P-value is less than 0.05.

## Discussion

The prognosis of patients with BMs is poor, with most patients dying within 6 months of receiving a BM diagnosis.^17^ The therapeutic options for BM are limited and the optimal BED for brain radiotherapy remains undetermined. This study is the first to our knowledge exploring and evaluating the optimal BED threshold for prognostic prediction for BM from SCLC. Our results indicated that administering a BED > 47.4 Gy to the brain resulted in significant prognostic benefits in terms of OS and BM-PFS, in patients with SCLC with BM. This finding held true regardless of BM number, clinical stage, and tumor size. Although there is a general lack of data from prospective randomized clinical trials to guide the implementation of a dose-escalation strategy for stereotactic radiotherapy based on BED in the management of patients with SCLC with BM, this study provides valuable information for clinicians that support the use of high-dose brain radiotherapy for BM.

Our study showed that use of high BED was effective in improving BM-PFS and OS among patients with SCLC with BM. We found that BED, smoking history, pleural effusion, and ECOG score may affect OS. Nevertheless, our results supported the establishment of general therapy guidelines for BM. For now, there is insufficient evidence to assert survival benefits of these treatments in clinical practice. However, our research suggests that high BED (>47.4 Gy) improves survival outcomes and decreases the likelihood of local recurrence.

Although numerous therapeutic options, including radiotherapy, radiosurgery, targeted therapy, surgery, chemotherapy, and immunotherapy, have been proposed for the management of advanced SCLC, WBRT remains the standard treatment for patients with BM from SCLC.^18^ The optimal radiation dose and fractions to be used for WBRT in patients with SCLC remain to be refined and standardized. Davey and Ennis^19^ reported improved control of BM in patients receiving 40 Gy of WBRT in 20 twice-daily fractions (BED = 48 Gy), as compared to patients receiving 20 Gy in 4-daily fractions (BED = 30 Gy). Vogel et al observed improved intracranial control in patients treated with WBRT after undergoing surgical resection of intracranial metastases. The median BED used for WBRT was 48.0 Gy, which is similar to the threshold used for BED in our study (BED = 47.4 Gy).^20^ In a randomized controlled trial reported by Andrews et al, a WBRT dose of 37.5 Gy in 15 fractions was delivered to patients with BM who were assigned to receive WBRT only; an additional boost dose of 15 to 24 Gy was delivered to patients assigned to receive WBRT plus an SRS boost. This study showed that WBRT plus an SRS boost (BED ≥ 39.06 Gy) was associated with better local control than that achieved with WBRT alone (BED = 37.5 Gy, *P* = .0132).^21^ Baliga et al^22^ conducted a meta-analysis by compiling data from 10 studies of patients receiving fractionated stereotactic radiotherapy for BM, regardless of primary tumor type. An improvement in local control associated with increasing BEDs was noted in patient with BM; for BEDs of 40, 50, and 60 Gy, the 1-year local control rates were 73%, 78%, and 84%, respectively; the 2-year local control rates were 62%, 69%, and 81%, respectively.^17^ Achieving local control is critical to prognosis and is generally associated with improved PFS and OS. In a study of 82 patients with SCLC with BM, Sun et al found that WBRT plus a radiation boost significantly prolonged survival as compared with WBRT only. However, the study failed to address the dose-escalation strategy and the contribution of BED to survival outcomes.^8^ Rodrigus et al retrospectively compared patients with NSCLC treated with 30 Gy/10 fractions WBRT plus a boost of 15 Gy/5 fractions (n = 62) with patients treated with 20 to 30 Gy/5 to 10 fractions WBRT only (n = 188). The results showed that treatment with a radiation boost significantly improved median survival to 8 months (*P* = .001).^23^ In agreement with these previous results, we observed a favorable prognostic value of BED > 47.4 Gy among patients with BM from SCLC. In patients treated with BED > 47.4 Gy, OS and 1-year survival rate were 17.5 months and 71.1%, respectively, significantly improved compared to those in patients treated with BED ≤ 47.4 Gy. The survival of patients treated with BED > 47.4 Gy was better than that reported previously by Chatani el al, who described survival rates of 6% and 4% at 1 year for patients with BM from lung adenocarcinoma treated with BED of 39.0 and 28.0 Gy WBRT, respectively.^24^

In this study, pleural effusion was found to be a prognostic factor that independently affected OS; patients without an initial diagnosis of pleural effusion had significantly improved OS. Although the prognostic effect and mechanism of pleural effusion in patients with BM from SCLC remain largely unknown,^25^ our results are consistent with those reported previously. Shojaee et al^26^ analyzed data from the Surveillance, Epidemiology, and End Results Program (SEER) and observed that patients with SCLC without pleural effusion had significantly better OS than those with pleural effusion (HR = 1.46, 95% CI: 1.41-1.50, *P* < .001). Morgensztern et al^27^ analyzed patients with NSCLC from the SEER database and determined that the presence of pleural effusion was associated with decreased median OS.

The ECOG performance status is a scale to assess patient’s ability to tolerate therapies and has been found to be a valuable indictor for survival prediction among patients with lung cancer.^28-32^ Leeward et al^33^ retrospectively reviewed 1292 patients with BM, typically from lung cancer, and reported survival rates of 32.0%, 39.0%, and only 10.0% at 1 year in patients with ECOG scores of 0, 1, and 3, respectively. Rades et al^34^ reported that ECOG scores of 0 to 1 were significantly associated with better survival (*P* < .001). In our study, the 1-year survival rates for patients with ECOG scores of 0 and 1 were 51.3% and 31.6%, respectively. Multivariate analysis confirmed that this survival disadvantage of higher ECOG performance status was independent of other major clinical and treatment factors affecting OS. Meanwhile, in our analysis, smoking history was also an independent predictor of OS, which is consistent with previous studies that have demonstrated the prognostic significance of tobacco smoke inhalation.^35,36^

Our study has some limitations. The study described a single-institution experience with 250 patients and was limited by its retrospective nature. Caution should be taken when generalizing the results to other populations. Second, this study evaluated prognosis among patients with BM from SCLC, without considering the possible therapeutic implications of SCLC molecular subtype. Third, toxicities related to radiation dose were not assessed to find the optimal dose for balancing the therapeutic and adverse effects. In the phase II clinical study of BM treated with hypofractionated stereotactic radiotherapy by Ernst-Stecken et al, BED at 5 × 6 to 7 Gy (equivalent to 40-49.58 Gy) was determined to be effective and safe for the treatment of BM.^37^

## Conclusions

Both BM-PFS and OS after brain radiotherapy were highly dependent on BED, supporting the use of BED-based brain radiotherapy as a promising strategy for the treatment of BM from SCLC. Use of a dose schedule with a BED of at least 47.4 Gy is recommended and is to be validated in large, prospective, and randomized clinical studies.
